# Update 2026: Management of Small Cell Lung Cancer

**DOI:** 10.1007/s00408-026-00927-6

**Published:** 2026-08-19

**Authors:** Mumtu Lalla, Puneet Dhillon, Buse Eglenen Polat, Haiying Cheng

**Affiliations:** Department of Medical Oncology, Montefiore Medical Center/Albert Einstein College of Medicine, Montefiore Einstein Comprehensive Cancer Center, Bronx, NY 10461 USA

**Keywords:** Small cell lung cancer, Tarlatamab, Durvalumab, Lurbinectedin, Antibody-drug conjugates, Molecular subtypes

## Abstract

Small cell lung cancer (SCLC) remains a biologically aggressive neuroendocrine carcinoma characterized by early metastatic spread, frequent relapse, and poor long-term survival. After several decades of limited progress, recent therapeutic advances have begun to reshape management across disease stages. In limited-stage SCLC, concurrent platinum-etoposide chemoradiotherapy remains the backbone of curative-intent treatment, while consolidation durvalumab after chemoradiotherapy has established a new standard for patients without disease progression. In extensive-stage SCLC, platinum-etoposide plus a PD-L1 inhibitor remains the preferred first-line approach. For selected patients, maintenance lurbinectedin plus atezolizumab and second-line tarlatamab have expanded treatment options beyond conventional cytotoxic chemotherapy. Intracranial management is also evolving, with greater emphasis on baseline brain magnetic resonance imaging, longitudinal surveillance, selective use of prophylactic cranial irradiation, stereotactic radiosurgery in appropriate patients, and neuroprotective strategies during whole-brain radiotherapy. Biologically, SCLC is increasingly recognized as a heterogeneous disease. Transcriptional subtypes, DLL3, B7-H3, SEZ6, SLFN11, and circulating tumor DNA are informing clinical trial design and may help refine future treatment selection. This review provides a clinically oriented overview of current standards and emerging therapeutic strategies in SCLC, with attention to evidence quality, toxicity, sequencing, and clinical applicability. It also discusses key unresolved questions that must be addressed before biomarker-directed and subtype-informed treatment can be integrated into routine practice.

## Background and Scope

Small cell lung cancer (SCLC) accounts for approximately 10–15% of all lung cancers and remains one of the most lethal thoracic malignancies, with survival gains lagging far behind those achieved in non-small cell lung cancer (NSCLC) [1–3]. Tobacco exposure remains the dominant risk factor, though occupational exposures, radon, chronic obstructive pulmonary disease (COPD) also contribute [4, 5]. Low-dose CT screening for eligible high-risk adults is less reliable for early SCLC detection given its short preclinical window and rapid metastatic potential [6].

SCLC is classified into limited (LS-SCLC) and extensive stage (ES-SCLC), linking disease extent to radiation feasibility and treatment intent. Approximately two-thirds of patients present with ES-SCLC, and most experience relapse despite initial chemosensitivity [2, 7]. Prognosis is shaped by stage, performance status, comorbidities, weight loss, lactate dehydrogenase (LDH), and the presence of brain or liver metastases. Socioeconomic and structural factors, including insurance status, race, and healthcare access, further drive outcome disparities and should inform how guidelines are applied in practice [7, 8].

This review focuses on practice-changing updates in SCLC management. It emphasizes evidence relevant to current clinical decision-making, including consolidation durvalumab in LS-SCLC, maintenance lurbinectedin plus atezolizumab in ES-SCLC, tarlatamab after platinum-based therapy, evolving intracranial management, and the emerging biomarker framework for future trials.

## Diagnostic Work-up and Staging

Most SCLC patients present clinically with cough, dyspnea, hemoptysis, weight loss, bulky mediastinal disease, metastatic symptoms, or paraneoplastic syndromes, including syndrome of inappropriate antidiuretic hormone secretion (SIADH) and Lambert-Eaten Myasthenic Syndrome [9]. Pathologic confirmation is essential. Classic morphology includes nuclear molding, high mitotic activity (Ki67 50–100%), and extensive necrosis [10]. Neuroendocrine markers, including synaptophysin, chromogranin A, CD56, and INSM1, support the diagnosis, although marker-negative tumors and crushed specimens can complicate interpretation [10, 11].

Baseline evaluation should proceed promptly, as delays can compromise outcomes. Contrast-enhanced CT of the chest, abdomen, and pelvis and brain magnetic resonance imaging (MRI) are central staging elements. Positron emission tomography (PET)/CT is useful when the results could change radiation fields or identify otherwise occult metastatic disease [11, 12]. Mediastinal staging with endobronchial ultrasound or mediastinoscopy is appropriate when nodal status affects radiation planning or surgical candidacy [13, 14]. TNM staging should be recorded in addition to the Veterans Administration Lung Study Group stage to improve prognostic precision and trial comparability [12, 15].

Molecular testing is not routine, given near-universal inactivation of TP53 and RB1 and rarity of actionable oncogenic drivers [16]. Next-generation sequencing or circulating tumor DNA (ctDNA) testing should nonetheless be considered in never-smokers, suspected combined histology, atypical clinical behavior, and clinical trial screening [17]. There are other unique SCLC subsets. Approximately 6–14% of SCLCs are RB proficient; these have a  unique p16-low/cyclinD1-high immunohistochemical profile, harbor CCND1 amplification (29%) and enriched in CDKN2A mutations (50%) and NSCLC-type alterations (KEAP1, STK11, FGFR1), and may harbor clinically actionable oncogenic drivers including KRAS G12C and novel fusions [18]. The application of ctDNA detection is also under investigation in SCLC. SCLC sheds high levels of ctDNA, and investigations have shown that variations may have clinical implications for outcomes. A study showed that in 81 SCLC patients with serial ctDNA levels collected at diagnosis, remission, and relapse, the median max VAF dropped from 53.3% → 0.15% → 38.65% across these timepoints. Patients with detectable residual RB1 alterations at completion of chemotherapy had a shorter DFS of 1 month vs 8 months (P = .02). This pointed to recommendation of early clinical trial enrollment in these high risk groups[19].

Treatment planning must define the intended goal before therapy begins: curative-intent chemoradiotherapy in LS-SCLC and disease control, symptom relief, and quality-of-life preservation in ES-SCLC. Pulmonary, marrow, and renal reserve, hearing, neuropathy, nutritional status, and social support should be assessed early, as these directly affect toxicity risk, radiation feasibility, and supportive-care planning. Functional assessment is equally important: poor performance status caused by tumor burden may improve rapidly with therapy, whereas poor status driven by frailty or comorbidity requires careful dose selection and early supportive-care integration [14, 20].

Proactive management of disease-and treatment-related complications is integral to care delivery. Tumor Lysis Syndrome can be clinically significant in SCLC given rapid chemosensitivity and warrants risk-stratified prophylaxis, aggressive electrolyte management, and early nephrology involvement. Paraneoplastic syndromes should be managed with syndrome-specific interventions and early specialist involvement. Supportive care is a cornerstone of comprehensive management and early palliative care integration, alongside nutritional and psychosocial support, physical therapy, palliative radiation, and bronchoscopic airway intervention can improve treatment tolerance and quality of life. Smoking cessation counseling should be additionally offered.

**Fig. 1:**
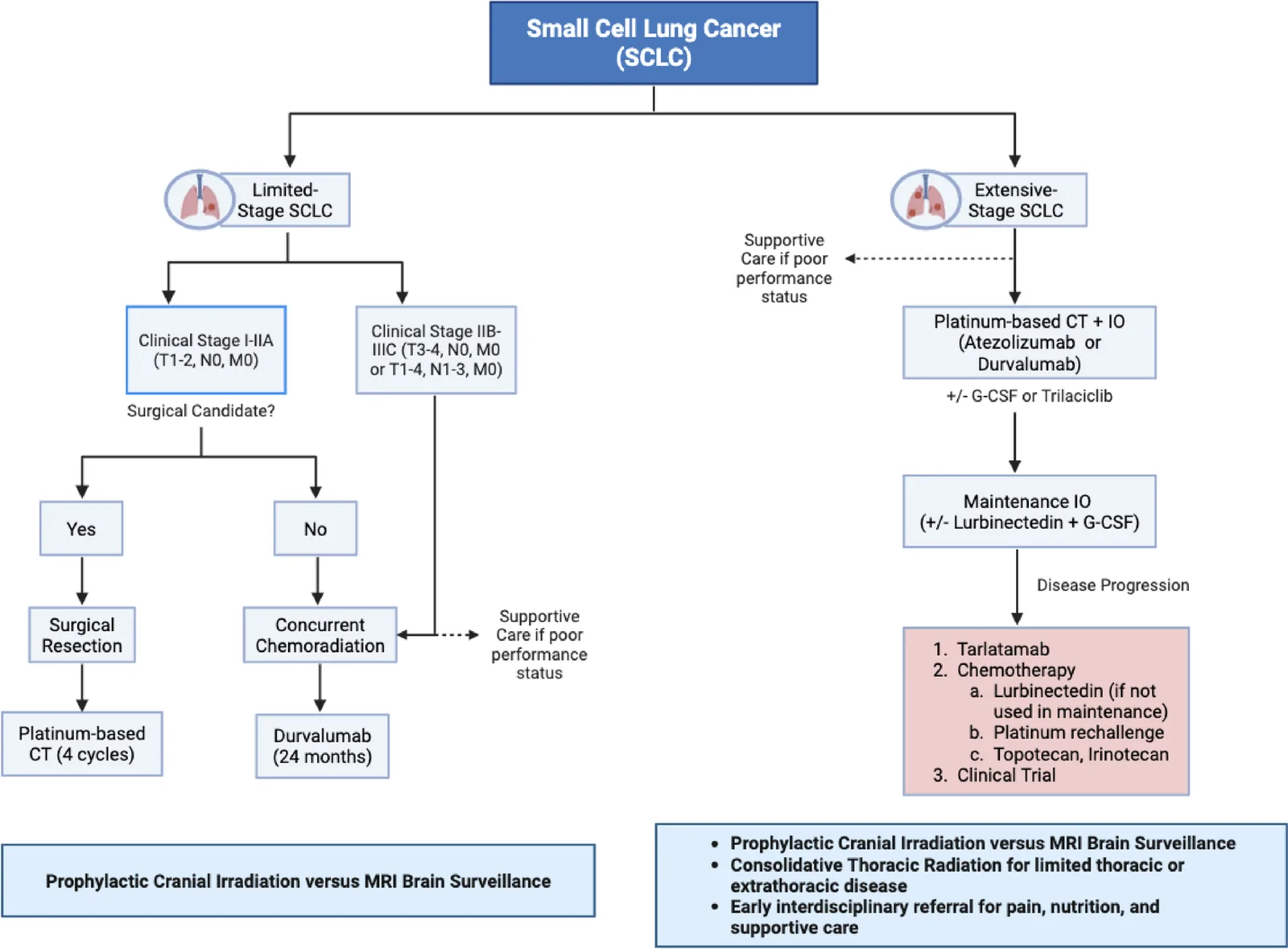
Treatment algorithm for SCLC. Created in BioRender. Lalla (2026) https://BioRender.com/q7wzub. CT, Chemotherapy; IO, immunotherapy

A treatment algorithm for LS-SCLC and ES-SCLC is summarized in Fig. 1.

## Limited-Stage SCLC (LS-SCLC)

### Concurrent Chemoradiation

Concurrent chemoradiation with platinum-etoposide remains standard for most patients with LS-SCLC [21, 22]. Cisplatin is commonly preferred, but carboplatin is reasonable when renal function, hearing loss, neuropathy, frailty, or tolerability is a concern; a large Veterans Affairs cohort found no significant survival difference between agents [23]. Adjunctive myeloid growth factors should be avoided during chemoradiation, as GM-CSF use in this context was associated with increased treatment-related mortality, severe thrombocytopenia, and nonhematologic toxicity in the phase III SWOG 8812 trial [24].

Concurrent chemoradiation is generally preferred over sequential approach when patients can tolerate it. JCOG 9104 showed longer median overall survival (mOS (27.2 vs. 19.7 months; *P* = 0.097) [12, 25], and meta-analyses support improved survival when the interval from therapy initiation to radiotherapy completion is shortened [26–28]. Sequential approach remains appropriate for older or frail patients or those with poor performance status, given the greater hematologic toxicity associated with concurrent delivery [25] .

The optimal dose and fractionation of radiation remain debated. Twice-daily (BID) radiation of 45 Gy in 30 fractions demonstrated the greatest efficacy in the landmark Intergroup 0096 trial, with improved OS over once-daily 45 Gy in 25 fractions (5-year OS 26% vs. 16%; *P* = 0.04) [29]. However, given the logistical burden of BID scheduling, once-daily radiation at higher doses (66–70 Gy) is an acceptable alternative, with the CONVERT and CALGB 30,610 trials showing comparable survival and toxicity to BID 45 Gy bid radiation [30, 31]. Higher-dose accelerated regimens, including 60 Gy in 40 BID fractions and 54 Gy SIB in 30 BID fractions, have additionally shown survival advantages without increased toxicity [32–34].

Definitive surgery is reserved for a small subset of patients presenting with clinical stage I–IIA (T1–2, N0, M0) disease. Lobectomy with mediastinal lymph node dissection is the preferred surgical approach and should be followed by four cycles of adjuvant chemotherapy [12].

### Consolidation Durvalumab

The approval of durvalumab consolidation following chemoradiation represents a transformative advance in LS-SCLC management. The phase 3 ADRIATIC trial demonstrated a significant OS benefit of durvalumab over placebo (55.9 months vs. 33.4 months, HR 0.73), with consistent benefit observed regardless of prior RT schedule, chemotherapy backbone, or PCI receipt [35]. A two-year duration of consolidative therapy is supported by the trial design, with a sustained PFS benefit evidenced throughout this period (24-month PFS 46.2% vs. 34.2%), reflecting the well-established pattern of early relapse in LS-SCLC [32]. Patients should be monitored for immune-related toxicities during and following treatment; pneumonitis, though uncommon, remains a relevant risk (3.1% durvalumab vs. 2.6 placebo) [35]. ADRIATIC stratified randomization by PCI receipt but was not powered for subgroup comparisons which the investigators state explicitly. Analysis supports that durvalumab should be offered regardless of whether PCI was given, and that omitting PCI does not appear to require withholding durvalumab [35]. An exploratory ASCO 2025 analysis by Barbie et al., compared early progressors (EP, PFS <6 mo) with long-term progression-free survivors (LTP, PFS >12 mo) in both arms using pre-cCRT tumor tissue [36]. There was a tendency of higher T-cell inflamed signature (TIS) and Stimulator of Interferon Genes (STING) pathway expression in LTPs within the durvalumab arm but not the placebo arm. The authors hypothesized that LTPs possessed a T-cell-inflamed microenvironment was more conducive to immunotherapy response [36]. DeLLphi-306 (NCT06117774) is an active Phase 3 trial that recruited patients with LS-SCLC who completed concurrent without progression then randomized to tarlatamab maintenance vs placebo. Co-primary endpoints are BICR-assessed PFS and OS. The results may be difficult to interpret since the comparator is placebo, not durvalumab, as the trial was designed before ADRIATIC established consolidation durvalumab as standard. Thus, it cannot establish superiority to durvalumab [37]. Additionally, placebo arm's post-progression treatments of chemoimmunotherapy and tarlatamab in the second line can confound any outcome differences. ADRIATIC investigators had noted that chemoimmunotherapy at relapse may have contributed to the unexpectedly positive placebo-arm median OS of 33.4 months [35]. Toxicities of a T-cell engager in a in a curative-intent, post-thoracic-RT population is not well established [38, 39]. To address the remaining questions, the ongoing PrE0512 trial (NCT07637513) is evaluating the efficacy of tarlatamab plus durvalumab compared with durvalumab alone. This open-label, randomized trial enrolls patients who have completed definitive chemoradiotherapy and randomizes them to receive consolidation therapy with either the combination of tarlatamab and durvalumab or durvalumab alone, and efficacy results are not yet available [40]. Adjuvant chemotherapy for resected LS-SCLC is typically recommended with adequate performance status; the established evidence base is a National Cancer Database cohort (adjuvant chemo HR 0.78, 95% CI 0.63–0.95; chemo + cranial RT HR 0.52, 95% CI 0.36–0.75) [41]. Currently, adjuvant durvalumab is supported exclusively in the primary chemoradiation setting, and there is no evidence to use it after resection [42]. There is an actively recruiting Alliance Foundation Trial (NCT07149363) phase 2, single-arm–design study trial studying consolidation therapy for completely resected small cell lung cancer. The intervention is adjuvant platinum-etoposide plus durvalumab in completely resected T1–T2 N0–1 M0 SCLC [43].

### Intracranial Management in LS-SCLC

Prophylactic Cranial Irradiation (PCI) historically reduced brain metastases and improved overall survival in LS-SCLC responding to chemoradiation [44, 45]. However, these studies predated routine baseline brain MRI staging, and subsequent analyses have not consistently confirmed an intracranial or survival benefit, particularly in pathologic stage I (T1-2, N0) disease [46, 47]. PCI is additionally associated with neurocognitive toxicity and functional decline and is generally not recommended for patients older than 60 years or those with a poor performance status or impaired baseline neurocognitive function [12]).

Current practice should individualize PCI versus MRI surveillance based on patient age, performance status, baseline cognition, disease stage, and patient preference [44, 48]. Brain MRI surveillance is recommended for all patients regardless of PCI status. If PCI is pursued, it should be administered prior to durvalumab initiation [35], and the addition of memantine and hippocampal avoidance techniques can be considered to mitigate neurocognitive decline (discussed further in Sect.  “Intracranial Radiation”).

## Extensive-Stage SCLC (ES-SCLC)

### First Line Induction Chemoimmunotherapy

Platinum-etoposide has served as the foundation of first-line therapy for ES-SCLC for more than two decades. Despite high initial response rates, early relapse is common and long-term survival remains poor [49].

The addition of immunotherapy to chemotherapy was the first major therapeutic breakthrough in ES-SCLC. Atezolizumab became the first immune checkpoint inhibitor approved in this setting based on the IMpower133 trial, which demonstrated significant improvements in both median progression-free survival (mPFS 5.2 vs. 4.3 months; HR 0.77) and OS (12.3 vs. 10.3 months; HR 0.70) with chemoimmunotherapy compared to chemotherapy alone [50, 51]. The CASPIAN study, which included patients with asymptomatic brain metastasis, subsequently led to the approval of durvalumab, showing similar improvements in mPFS and mOS with chemoimmunotherapy over chemotherapy alone (mOS 13.0 vs. 10.3 months; HR 0.73) [52].

Building on these PD-L1 inhibitor approvals, parallel trials evaluated the PD-1 inhibitors pembrolizumab and nivolumab but failed to achieve a significant OS benefit, precluding their approval. In contrast, serplulimab, another PD-1 inhibitor, was approved in China based the ASTRUM-005 trial, which showed significant improvements in mPFS (5.7 vs. 4.3 months; HR 0.48) and mOS (15.4 vs. 10.9 months; HR 0.63) with chemoimmunotherapy compared to chemotherapy alone [53]. Notably, serplulimab demonstrated a numerically superior OS relative to atezolizumab and durvalumab, a difference that may reflect its increased affinity for PD-1, its capacity to block both PD-L1 and PD-L2 signaling, the predominantly Asian population enrolled, or the higher proportion of non-smokers in the study, though cross-trial comparisons should be interpreted with caution. The accruing ASTRIDE trial (NCT05468489) will compare serplulimab with atezolizumab in first-line combination chemoimmunotherapy for ES-SCLC.

The PD-L1 inhibitor adebrelimab and the PD-1 inhibitors toripalimab and tislelizumab have additionally been approved in China based on the CAPSTONE-1, RATIONALE-31, and EXTENTORCH studies, respectively [54–56]. A meta-analysis of randomized clinical trials suggests comparable efficacy of PD-1 and PD-L1 inhibitors, but a lower incidence of grade ≥3 adverse events (AE), any-grade immune-related adverse events, and grade ≥3 immune-related adverse events with PD-L1 inhibitors (*p* < 0.01) [57].

First-line chemoimmunotherapy causes toxicity from both chemotherapy and immune checkpoint blockade, though the incidence of grade 3–4 adverse events is comparable between chemoimmunotherapy and chemotherapy alone in pivotal trials [50, 52]. Chemotherapy-related myelosuppression is the dominant toxicity, with neutropenia being the most frequent AE; fatigue, nausea/vomiting, and alopecia are also clinically significant. The addition of immunotherapy introduces the risk of irAEs: while rash and hypothyroidism are the most common, pneumonitis, hepatitis, and colitis are rare but potentially severe complications.

Adjunctive supportive care strategies should be considered to mitigate these toxicities. G-CSF or trilaciclib can be used alongside chemoimmunotherapy to prevent myelosuppression [12]. Trilaciclib, a CDK 4/6 inhibitor, provides myeloprotection by inducing transient cell cycle arrest of hematopoietic stems cells, and has been shown to reduce the incidence of severe neutropenia, neutropenic fever, anemia, and thrombocytopenia [58, 59]. Trilaciclib administration should be particularly considered in patients anticipated for maintenance intensification with lurbinectedin. A three-drug antiemetic regimen is also essential for prevention of chemotherapy-induced emesis.

### Maintenance Strategies

Although most ES-SCLC tumors are initially platinum-sensitive, responses seldom durable even after chemoimmunotherapy [50, 52]. Maintenance intensification has consequently emerged as a strategy to prolong the response to first-line treatment (Table 1).

The IMpower133 and CASPIAN trials established maintenance immunotherapy as standard practice in ES-SCLC, where patients without diseaseithout progressionion after induction chemoimmunotherapy are continued on maintenance immunotherapy until progression or intolerable toxicity [50, 52]. Although no randomized trial has been performed to quantify this benefit, a post-hoc analysis of IMpower133 demonstrated significant improvements in mPFS (2.6 vs. 1.8 months; HR 0.63) and mOS (12.5 vs. 8.4 months; HR 0.59) with maintenance atezolizumab versus placebo [60].

The IMforte trial investigated the addition of lurbinectedin, a synthetic alkaloid, to immunotherapy maintenancewho were fit (Eastern Cooperative Oncology Group, ECOG, performance status 0–1) and without brain metastases were randomized to lurbinectedin plus atezolizumab or atezolizumab alone [61, 61]. Compared to atezolizumab alone, the combination demonstrated significant improvements in mPFS (5.4 vs. 2.7 months; HR 0.54) and mOS (13.2 vs. 10.6 months; HR 0.73), measured from randomization at the start of maintenance,. Patients without disease progression after induction chemoimmunotherapy [61]. As anticipated with chemotherapy-based intensification, hematologic toxicity was higher in the combination arm [61]. G-CSF prophylaxis is recommended to minimize neutropenic fever, and trilaciclib may additionally be considered to minimize myelosuppression [12]. Practically, this regimen is preferable for patients that are fit, tolerated induction without significant myelosuppression and toxicity, and do not have brain metastasis, given the limited CNS penetration of lurbinectedin.

Early-phase data have also suggested promise for maintenance intensification with tarlatamab, a DLL3-directed bispecific T-cell engager (BiTE). Informed by preclinical data indicating that tarlatamab upregulates PD-L1 expression and demonstrates therapeutic synergy with PD-L1 inhibition, the phase 1b DeLLphi-303 study evaluated the combination of tarlatamab and a PD-L1 inhibitor (atezolizumab or durvalumab) in patients who received induction chemoimmunotherapy without progression [62, 63]. At a median follow-up of 18.4 months, the combination yielded a median duration of response (DOR) of 16.6 months and a mOS of 25.3 months [63]. The safety profile was consistent with tarlatamab monotherapy (as discussed below), with cytokine release syndrome (CRS; 24%), pyrexia (7%), and immune effector cell-associated neurotoxicity syndrome (ICANS; 5%) being the most common serious side effects [63]. CRS was predominantly grade 1 and did not lead to any tarlatamab discontinuations [63]. The phase 3 DeLLphi-305 trial (NCT06211036) is currently underway  to compare the efficacy of tarlatamab plus durvalumab against durvalumab alone. Additionally, the phase 3 DeLLphi-312 (NCT07005128) is evaluating tarlatamab in both the first-line induction and maintenance settings. Novel maintenance approaches are also being investigated, incorporating biomarker-based strategies (discussed further below) and alternative therapeutic targets. The phase II SWOG S1929 study evaluated the combination of the poly (ADP-ribose) polymerase (PARP) inhibitor talazoparib and atezolizumab in patients with Schlafen 11 (SLFN11)-positive ES-SCLC, finding an improved mPFS (2.9 versus 2.4 months, HR 0.66, 80% confidence interval: 0.50–0.86) with the combination versus atezolizumab alone, though no difference in OS was observed [64, 65]. Although not practice changing, this trial provided proof of concept for further biomarker-directed studies in this space [64]. The phase Ib/II Ideate-Lung03 trial (NCT06362252) is currently evaluating the B7-H3 antibody drug conjugate (ADC, discussed further below) ifinatamab deruxtecan (I-DXd) in the first-line induction or maintenance settings.

**Table 1:** First-line and maintenance treatment options for extensive-stage small cell lung cancer

Trial	Regimen	Outcomes (mOS in mo)	FDA approved
IMpower133	Atezolizumab + carboplatin/etoposide	12.3 vs. 10.3 (HR 0.70)	Yes (2019)
CASPIAN	Durvalumab + cisplatin or carboplatin/etoposide	mOS: 13.0 vs. 10.3 (HR 0.73)	Yes (2020)
KEYNOTE-604	Pembrolizumab + cisplatin or carboplatin/etoposide	No significant benefit in mOS	No
ASTRUM-005	Serplulimab + carboplatin/etoposide	15.4 vs. 10.9 (HR 0.63)	No (US)
CAPSTONE-1	Adebrelimab + carboplatin/etoposide	15.3 vs. 12.8 (HR 0.72)	No (US)
CheckMate 451	Nivolumab ± ipilimumab maintenance (after platinum/etoposide)	No significant benefit in mOS	No
IMforte	Lurbinectedin + atezolizumab maintenance (after atezolizumab + carboplatin/etoposide induction)	13.2 vs. 10.6 (HR 0.73)	Yes (2025)

Trial	Maintenance regimen	Outcomes (mOS in mo)	FDA approved	References
IMforte	Lurbinectedin + atezolizumab	13.2 vs. 10.6 (HR 0.73)	Yes (2025)	[61]
CASPIAN	Durvalumab + cisplatin or carboplatin/etoposide	mOS: 13.0 vs. 10.3 (HR 0.73)	Yes (2020)	[63]
KEYNOTE-604	Pembrolizumab + cisplatin or carboplatin/etoposide	No significant benefit in mOS	No	[65]
ASTRUM-005	Serplulimab + carboplatin/etoposide	15.4 vs. 10.9 (HR 0.63)	No (US)	
CAPSTONE-1	Adebrelimab + carboplatin/etoposide	15.3 vs. 12.8 (HR 0.72)	No (US)	
	Nivolumab ± ipilimumab maintenance (after platinum/etoposide)	No significant benefit in mOS	No	

### Second-Line Therapy

Tarlatamab has changed the treatment landscape after platinum-based therapy. The phase III DeLLPhi-304 trial, which compared tarlatamab to chemotherapy (topotecan, lurbinectedin, amrubicin), showed superior mPFS (5.3 vs. 4.3 months; HR 0.71) and mOS (13.6 vs. 8.3 mo; HR 0.6) with tarlatamab [38]. Meaningful intracranial activity has also been demonstrated in the phase 1 DeLLphi-301 trial and real-world studies, supporting omission of brain radiation in select patients receiving tarlatamab [66–68].

Despite its efficacy, tarlatamab carries a distinctive toxicity profile driven by its mechanism of action as a BiTE. CRS and ICANS are the most clinically significant adverse effects, necessitating a step-up dosing strategy with 22–24 h monitoring in appropriate healthcare setting for the first two infusions (Cyle 1 Day 1 and Day 8) [12]. In the DeLLphi-304 trial, CRS occurred in 56% of patients, with the majority being grade 1 (42%) and occurring after the first two tarlatamab doses; most resolved with supportive care alone, though 16% required glucocorticoids and 4% required tocilizumab [38, 54]. ICANS was less frequent, occurring in 6% of patients and predominantly grade 1–2 [38]. Real-world data suggest higher rates of both CRS (70–80%) and ICANS (40–45%) [66, 68], underscoring the need for further studies to define risk factors and evaluate prophylactic strategies. Emerging real-world data from small single-center studies support prophylactic tocilizumab use in high-risk patients to decrease the incidence of high-grade CRS [69, 70]. Further studies are needed to define predictive biomarkers for response to tarlatamab, identify risk factors for CRS and ICANS, and evaluate eligibility for prophylactic strategies.

Access to tarlatamab is further limited by its cost, the current requirement for inpatient monitoring, and the multidisciplinary education and care coordination required for administration and toxicity management [71]. Patients treated in the real-world setting also often have worsen performance status and greater comorbidities than clinical trial populations, raising concern for toxicity management in the outpatient setting. Standardized toxicity grading, well-defined staffing workflows for toxicity mitigation, and prophylactic tocilizumab use may increase feasibility of transitioning tarlatamab administration to the outpatient setting.

For patients who are not candidates for tarlatamab, chemotherapy remains an important alternative. Patients with platinum-sensitive disease (relapse more than 6 months after platinum-based induction), may be considered for platinum-rechallenge, which demonstrated improved overall response rate (ORR 49% vs. 25%), mPFS (4.7 vs. 2.5 months), and tolerability compared to topotecan [72]. Lurbinectedin, irinotecan and topotecan remain additional alternatives [73](Table 2). Enrollment in clinical trials should be encouraged whenever feasible.

**Table 2:** Second-line and subsequent treatment options for extensive stage small cell lung cancer

Trial	Regimen	Median OS (mo)	OS HR (95% CI)	ORR	FDA approved
DeLLphi-304	Tarlatamab 10 mg IV Q2W vs. topotecan/lurbinectedin/amrubicin	13.6 vs. 8.3	0.60 (0.47–0.77)	35% vs. 20%	Yes (2024, accelerated; confirmed 2025)
Lurbinectedin phase 2 basket	Lurbinectedin 3.2 mg/m^2^ IV Q3W (single-arm)	9.3	–	35%	Yes (accelerated, 2020)
ATLANTIS	Lurbinectedin + doxorubicin vs. topotecan/CAV	8.6 vs. 7.6	0.97 (0.82–1.15)	32% vs. 29%	No (combo not approved)
Topotecan vs. CAV	Topotecan vs. cyclophosphamide/doxorubicin/vincristine	25 vs. 24.7 wk	NS	24% vs. 18%	Yes (topotecan)
Topotecan vs. BSC	Oral topotecan vs. best supportive care	25.9 vs. 13.9 wk	–	7%	Yes (topotecan)
French phase 3 (platinum rechallenge)	Carboplatin/etoposide vs. topotecan (sensitive relapse)	7.5 vs. 7.4	NS	49% vs. 25%	–
JCOG0605	Cisplatin/etoposide/irinotecan vs. topotecan (sensitive relapse)	18.2 vs. 12.5	–	84% vs. 27%	–
ACE Phase 3	Amrubicin vs. topotecan	7.5 vs. 7.8	0.88 (NS)	31% vs. 17%	Japan only

### Emerging Subsequent-Line Treatments

Third line and subsequent treatments are limited and associated with poor outcomes. Novel therapeutic agents targeting B7 homolog 3 (B7-H3) and seizure-related homolog protein 6 (SEZ6) are emerging, with promising efficacy observed in early-phase trials.

B7-H3 is a transmembrane protein moderately to highly expressed in SCLC, with low or absent expression in normal tissues [74]. Ifinatamab deruxtecan (I-DXd) is a first-in-class antibody drug conjugate (ADC) comprised of an anti-B7-H3 monoclonal antibody, a tetrapeptide-based cleavable linker, and a topoisomerase I inhibitor payload. In the Phase II IDeate-Lung01 trial, I-DXd demonstrated an ORR of 48.2%, mPFS of 4.9 months, and mOS of 10.3 months in previously treated ES-SCLC patients [75]. Subgroup analysis revealed an enriched ORR among patients previously treated with DLL3-targeted BiTEs, and comparable ORR across patients previously treated with PD-(L)1 inhibitors, lurbinectedin, and topoisomerase I inhibitors [75]. Intracranial ORR was 46.2% among patients with baseline brain metastases [75]. Consistent with other topoisomerase I-based ADCs, the most common AEs with I-DXd were nausea (43.1%), anemia (34.3%), and neutropenia (34.3%) [75]. Treatment-related interstitial lung disease (ILD), a known class effect of ADCs, occurred in 12.4% of patients, with 4% being grade ≥ 3 [75]. The phase III IDeate-Lung02 trial (NCT06203210), comparing I-DXd to chemotherapy (topotecan, amrubicin, or lurbinectedin) in patients with relapsed SCLC, is currently underway.

YL201 is a novel B7-H3 ADC that combines a protease-cleavable linker that enables dual payload release, extracellularly in the tumor microenvironment and intracellularly within lysosomes, with a topoisomerase 1 inhibitor [76]. In a phase 1/1b trial across advanced solid tumors, YL201 demonstrated an ORR of 63.9%, mPFS of 6.3 months, and median DOR of 5.7 months in 72 patients with ES-SCLC [76]. Hematologic toxicities were the most common AEs but the incidence of ILD was low (1%) [76]. A phase III trial (NCT06612151) comparing YL201 to topotecan in relapsed ES-SCLC is currently open.

SEZ6 is cell-surface protein normally expressed in the brain and eye that is aberrantly overexpressed in SCLC [74]. ABBV-706, a next-generation SEZ6-targeted ADC with a topoisomerase I payload, is currently under evaluation in a phase I trial (NCT05599984), with preliminary results showing an ORR of 58% in 80 patients with relapsed/refractory SCLC and enhanced efficacy in topoisomerase-I-naïve and second-line patients [77]. The toxicity profile is consistent with other topoisomerase I-based ADCs, with anemia (54%), neutropenia (27%), and thrombocytopenia (41%) being the most common and ILD observed in 11% of patients [77]. The Phase II SEZanne trial (NCT07155174) is currently open to evaluation ABBV-706 in combination with atezolizumab in previously untreated ES-SCLC.

### Radiation Updates in ES-SCLC

#### Consolidative Thoracic Radiotherapy

Consolidative thoracic radiotherapy (RT) is considered for select patients achieving a complete or partial response to systemic therapy, particularly those with residual thoracic disease and low-volume extrathoracic disease (NCCN category 2 A) [12]. Three randomized trials [78–80] support this approach, with improvements in PFS (HR 0.72, *P* < 0.001) and intrathoracic progression (relative risk 0.52) [81]. Although these trials predate immunotherapy, the benefit of consolidative RT has been extrapolated to contemporary practice, albeit with limited data on optimal sequencing or safety [82]. Radiation dosing should be individualized, with higher doses (45–54 Gy) favored in patients with a longer life expectancy [12]. The role of thoracic RT in the context of chemoimmunotherapy is under prospective evaluation in the RAPTOR/NRG LU007 (NCT04402788) and RISE (NCT06223711) studies.

#### Intracranial Radiation

The role of PCI in ES-SCLC has evolved considerably. Current NCCN guidelines prioritize MRI surveillance, listing PCI only as a consideration in selected patients [12]. PCI was previously considered standard of care following an EORTC (European Organization for Research and Treatment of Cancer) trial that demonstrated significant improvements in median disease-free survival (12.0 vs. 14.7 weeks, HR 0.76) and OS (5.4 vs. 6.7 months, HR 0.68) [83]. However, a critical limitation of the trial was the absence of baseline brain imaging, raising concern for undetected brain metastasis at enrollment. A large meta-analysis of 26,467 patients with ES-SCLC failed to demonstrate an OS benefit from PCI in patients without baseline brain metastasis (HR 0.74; *p* = 0.08) [48], and a phase III Japanese study similarly found no survival advantage with PCI over close MRI surveillance (11.6 vs. 13.7 months, HR 1.27, *p* = 0.094), supporting the current preference for MRI surveillance [84]. The ongoing SWOG S1827/MAVERICK trial (NCT04155034) is prospectively evaluating whether MRI surveillance is noninferior to MRI surveillance combined with PCI.

Whole-brain radiation therapy (WBRT) has been the conventional treatment of brain metastasis in ES-SCLC, though stereotactic radiosurgery (SRS) has emerged as a preferential approach for patients with a limited number of lesions [12]. The FIRE-SCLC cohort study showed that although central nervous system progression rates were higher with SRS than WBRT (HR 0.37, *p* < 0.001), OS favored SRS (8.5 months vs. 5.2 months, *p* < 0.001) [85]. A meta-analysis of 18,130 patients similarly demonstrated longer OS with SRS compared with WBRT, with or without an SRS boost (HR 0.85) [86]. A recent prospective phase II trial evaluating first-line SRS in patients with 1–10 brain metastasis reported a neurologic death rate of 11% at one year, compared with 17.5% in historical WBRT data, and mOS of 10.2 months; notably; 76% of patients avoided WBRT throughout their clinical course [87]. The NRG-CC009 (NCT04804644) is currently enrolling to compare SRS with hippocampal-sparing WBRT in patients with 1–10 brain metastases.

Neurocognitive toxicity from cranial irradiation can be mitigated with hippocampal-avoidance and memantine. The RTOG 0614 trial demonstrated that memantine prolonged time to cognitive decline without added toxicity compared to placebo (HR 0.78, *p* = 0.01) [88]. The phase III NRG CC001 trial further exhibited that hippocampal-avoidant WBRT (HA-WBRT) plus memantine reduced the risk of cognitive failure compared with WBRT and memantine (HR 0.74; *P* = 0.02), without differences in PFS and OS [89]. Together, these data support HA-WBRT with memantine as the standard approach to minimize neurocognitive toxicity during brain irradiation.

## Biology Subtypes and Biomarkers

### Genomic Landscape of SCLC

SCLC has historically been considered a homogeneous pathologic entity, genomically defined by the near-universal biallelic inactivation of *TP53* and *RB1* [16]. Despite these canonical alterations, SCLC lacks reliable predictive biomarkers to guide treatment selection. Conventional immune biomarkers, including PD-L1 expression and tumor mutational burden, are not reliably predictive of immunotherapeutic response, and actionable oncogenic drivers are rare [90, 91].

### Molecular Subtypes and Clinical Translation

Transcriptomic profiling has reframed SCLC as a molecularly heterogeneous disease and defined four major SCLC subtypes [92, 93]. SCLC-A, SCLC-N, and SCLC-P are enriched for ASCL1, NEUROD1, and POU2F3, respectively. SCLC-I has low expression of these transcription factors and an inflamed gene signature with increased immune checkpoint expression and immune-cell infiltration [92]. SCLC-A and SCLC-N are the most prevalent and are typically associated with high neuroendocrine marker expression, whereas SCLC-P and SCLC-I exhibit lower neuroendocrine differentiation [79]. Although intra- and intertumoral subtype heterogeneity exists, most tumors can be assigned to a single predominant subtype [92].

**Fig. 2:**
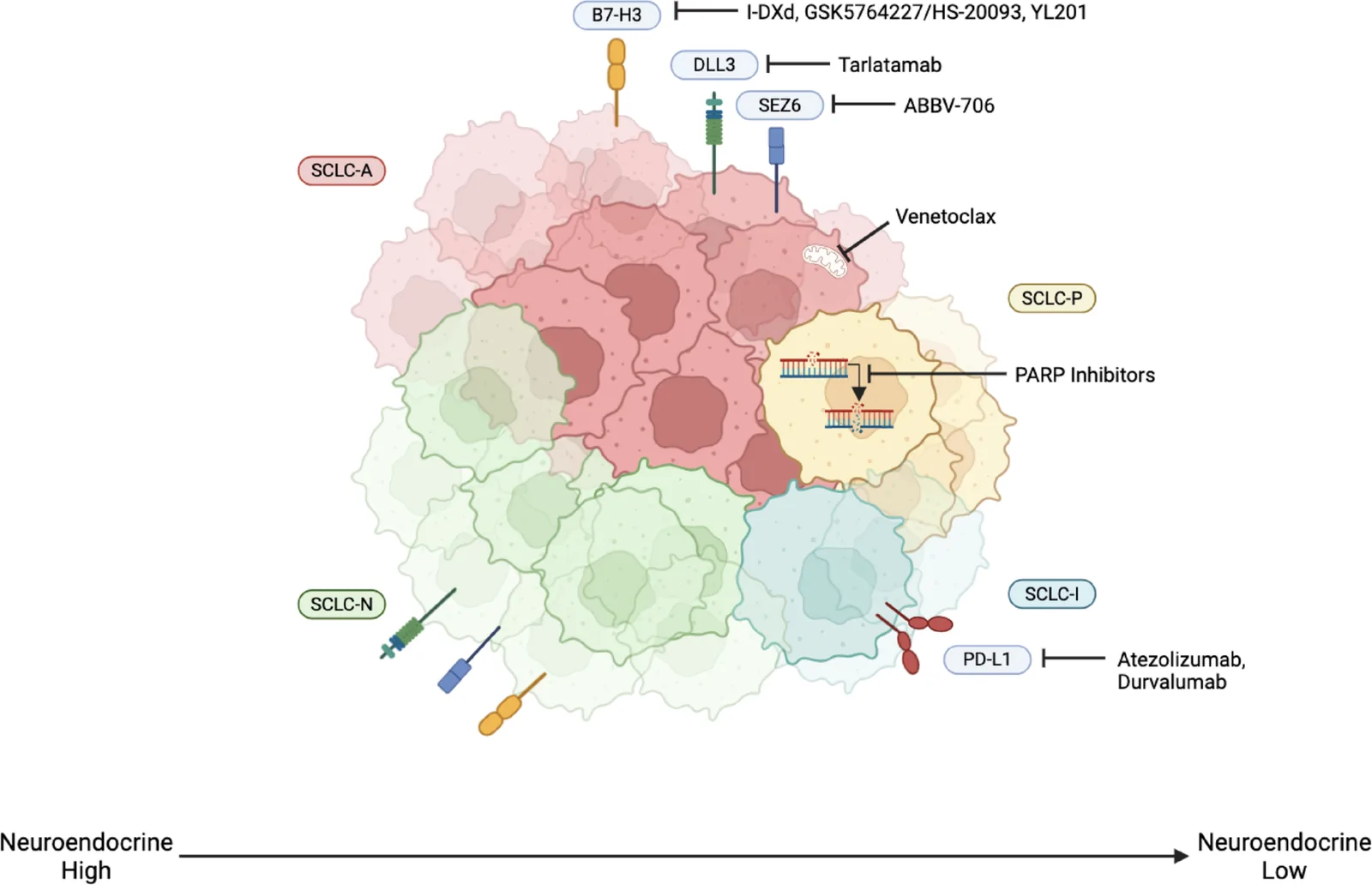
Molecular subtypes of SCLC and associated therapeutic vulnerabilities

**Fig. 3:**
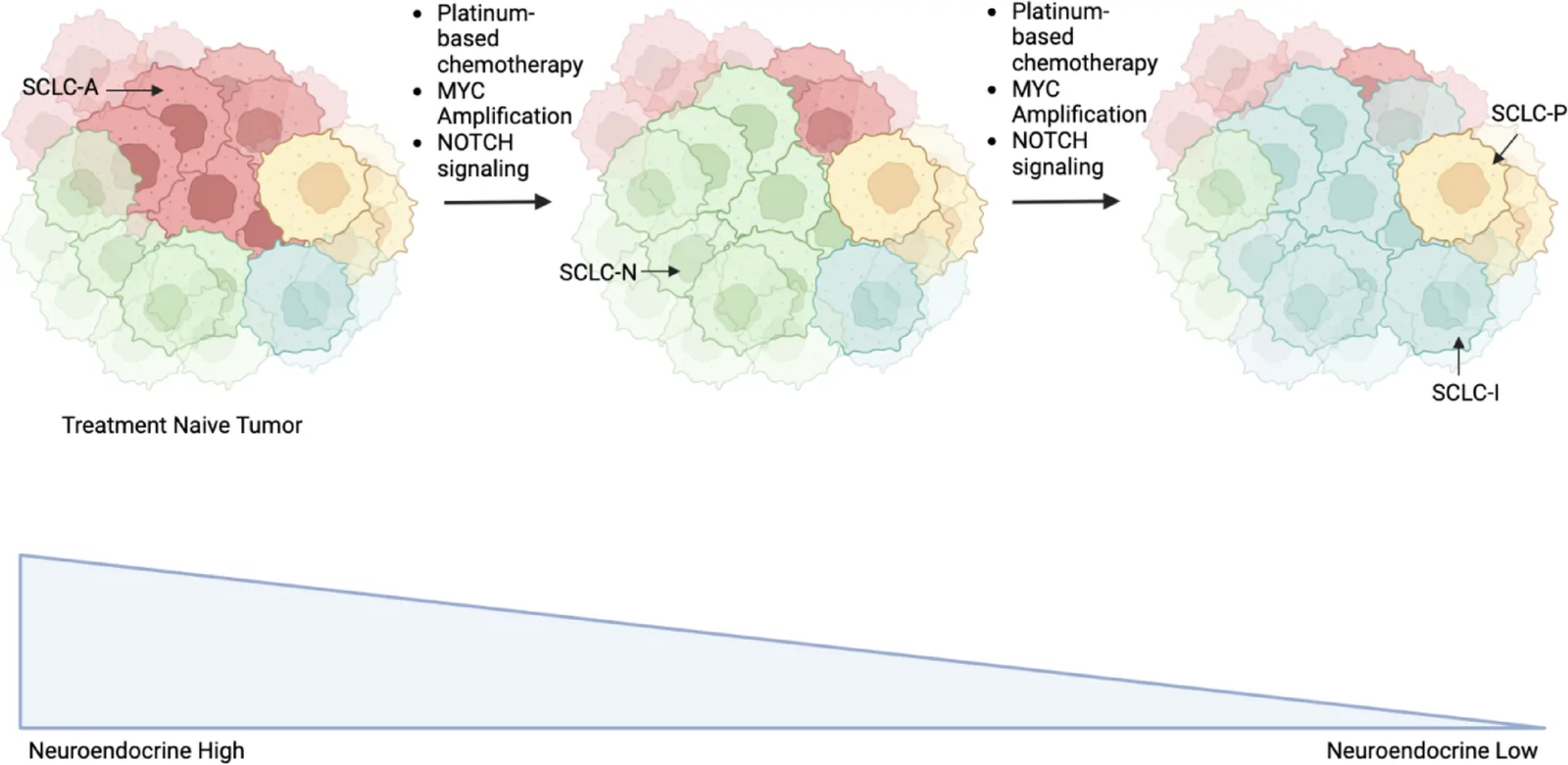
Phenotypic plasticity of SCLC

These subtypes are associated with distinct therapeutic vulnerabilities, providing a framework for personalized treatment selection and sequencing (Fig. 2). Subtype-stratified analysis of the IMpower133 and CASPIAN long-term outcomes demonstrated that the addition of immunotherapy to chemotherapy confers the greatest benefit in SCLC-I tumors [94, 95, 74]. Subtype-specific variation also exists in the expression of novel therapeutic targets: DLL3 and SEZ6 expression are highest in SCLC-A and lowest in SCLC-P, whereas B7-H3 expression is more uniformly distributed [74]. Preclinical studies have further illuminated subtype-specific vulnerabilities that may inform new therapeutic strategies (Fig. 2). Increased expression of schlafen-11 (SLFN11), a protein associated with DNA repair, has been observed in both SCLC-A and SCLC-N and is associated with enhanced susceptibility to DNA-damaging agents, including PARP inhibitors and platinum-based chemotherapy [65, 96]. The high intrinsic replication rates in these neuroendocrine subtypes also confers reliance on DNA damage response genes, predicting sensitivity to ATR (ataxia telangiectasia and Rad-3-related) inhibitors, particularly in SLFN11-negative tumors [84, 97–99]. Building on these observations, the PRISM/S2409 (NCT06769126) clinical trial is evaluating biomarker-driven treatment selection to guide first-line maintenance therapy in ES-SCLC, representing a proof-of-concept for subtype-informed precision medicine. Despite these advances, clinical implementation of subtype-driven approaches is challenged by the dynamic biology of SCLC. Phenotypic plasticity, the capacity to transition between molecular states under therapeutic pressure, is increasingly recognized as central driver of treatment resistance (Fig. 3) [92]. Emerging data suggests that treatment can trigger a shift from neuroendocrine (SCLC-A and SCLC-N) to non-neuroendocrine subtypes (SCLC-I) (Fig. 3) [99]. A deeper understanding of the mechanisms governing subtype plasticity may ultimately enable more effective therapeutic sequencing and combination strategies, allowing for more durable clinical benefit.

Phenotypic plasticity, the capacity to transition between molecular states under therapeutic pressure, is increasingly recognized as central driver of treatment resistance (Fig. 3) [92]. A deeper understanding of the mechanisms governing subtype plasticity may ultimately enable more effective therapeutic sequencing and combination strategies.

The four transcriptional subtypes of SCLC harbor distinct therapeutic vulnerabilities. Neuroendocrine-high subtypes (SCLC-A and SCLC-N) exhibit elevated DLL3 and SEZ6 expression and increased SLFN11 expression, conferring susceptibility to DNA-damaging agents including PARP inhibitors. SCLC-A is additionally enriched for BCL2, regulated by ASCL1, making it a candidate for BCL2 inhibition with venetoclax. SCLC-I is characterized by an inflamed gene signature and derives the greatest benefit from immunotherapy. Created in BioRender. Lalla (2026) https://BioRender.com/iue83z3.

SCLC is characterized by plasticity or the dynamic ability to switch molecular subtypes during treatment. Therapeutic pressure can trigger MYC amplification and downstream NOTCH signaling, resulting in the shift from a neuroendocrine high to a neuroendocrine low subtype. In contrast, SCLC-P is a more discrete subtype and may demonstrate less plasticity. Created in BioRender. Lalla (2026) https://BioRender.com/oi0zwla.

## Conclusions and Future Directions

SCLC management has changed substantially despite the disease’s aggressive biology. In LS-SCLC, concurrent chemoradiotherapy followed by consolidation durvalumab has established a new standard for appropriate patients without progression [32]. In ES-SCLC, platinum-etoposide plus a PD-L1 inhibitor remains the first-line foundation, while maintenance lurbinectedin plus atezolizumab and second-line tarlatamab have expanded options beyond conventional chemotherapy [38, 50, 52, 61]. Looking ahead, a growing pipeline of novel ADCs targeting emerging therapeutic targets, including B7-H3 and SEZ6, holds further promise, though optimal sequencing remains undefined [75, 100].

As novel agents enter the field, multidisciplinary care, toxicity expertise, access to specialized monitoring, and real-world data will be essential to preserve quality of life and reduce disparities. Future progress will depend on prospective biomarker validation and better sequencing. Key questions include which patients benefit most from maintenance intensification, when DLL3-directed therapy should be introduced, how ADCs should be combined or sequenced, and whether transcriptional subtype assignment can be implemented reliably in routine practice [92, 93]. Ultimately, durable improvement in outcomes will require trials that reflect the real clinical features of SCLC, including rapid progression, CNS involvement, frailty, and limited access to later-line therapy.

## Data Availability

No datasets were generated or analysed during the current study.

## Abbreviations

ADC: Antibody drug conjugate
AE: Adverse event
AJCC: American Joint Committee on Cancer
ATR: Ataxia telangiectasia and Rad-3-related
B7-H3: B7 homolog 3
BiTE: Bispecific T-cell engager
CRS: Cytokine release syndrome
ctDNA: Circulating tumor DNA
DOR: Duration of response
ECOG: Eastern Cooperative Oncology Group
EORTC: European Organization for Research and Treatment of Cancer
ES-SCLC: Extensive-stage small cell lung cancer
HA-WBRT: Hippocampal avoidant whole brain radiation therapy
ICANS: Immune effector cell-associated neurotoxicity syndrome
I-DXd: Ifinatamab deruxtecan
INSM1: Insulinoma-associated protein 1
irAE: Immune-related adverse event
ILD: Interstitial lung disease
LDH: Lactate dehydrogenase
LS-SCLC: Limited-stage small cell lung cancer
m PFS: Median progression-free survival
mOS: Median overall survival
MRI: Magnetic resonance imaging
NCCN: National comprehensive cancer network
ORR: Overall response rate
OS: Overall survival
PCI: Prophylactic cranial irradiation
PET: Positron emission tomography
PS: Performance status
RT: Radiation therapy
SLFN11: Schlafen-11
SCLC: Small cell lung cancer
SEZ6: Seizure-related homolog protein 6
SIADH: Syndrome of inappropriate antidiuretic hormone secretion
SRS: Stereotactic radiosurgery
WBRT: Whole-brain radiation therapy
